# Ultrafast Laser-Based Spectroscopy and Sensing: Applications in LIBS, CARS, and THz Spectroscopy

**DOI:** 10.3390/s100504342

**Published:** 2010-04-29

**Authors:** Megan R. Leahy-Hoppa, Joseph Miragliotta, Robert Osiander, Jennifer Burnett, Yamac Dikmelik, Caroline McEnnis, James B. Spicer

**Affiliations:** 1 Milton S. Eisenhower Research Center, The Johns Hopkins University Applied Physics Laboratory, 11100 Johns Hopkins Road, Laurel, MD 20723, USA; E-Mails: joseph.miragliotta@jhuapl.edu (J.M.); robert.osiander@jhuapl.edu (R.O.); 2 Department of Physics and Astronomy, The University of Louisville, KY, USA; E-Mail: jennifer.burnett@louisville.edu; 3 Department of Electrical and Computer Engineering, The Johns Hopkins University, Baltimore, MD, USA; E-Mail: yamac@jhu.edu; 4 Department of Materials Science and Engineering, The Johns Hopkins University, Baltimore, MD, USA; E-Mails: mcennis@jhu.edu (C.M.); spicer@jhu.edu (J.B.S.)

**Keywords:** laser-induced breakdown spectroscopy, Raman spectroscopy, terahertz spectroscopy

## Abstract

Ultrafast pulsed lasers find application in a range of spectroscopy and sensing techniques including laser induced breakdown spectroscopy (LIBS), coherent Raman spectroscopy, and terahertz (THz) spectroscopy. Whether based on absorption or emission processes, the characteristics of these techniques are heavily influenced by the use of ultrafast pulses in the signal generation process. Depending on the energy of the pulses used, the essential laser interaction process can primarily involve lattice vibrations, molecular rotations, or a combination of excited states produced by laser heating. While some of these techniques are currently confined to sensing at close ranges, others can be implemented for remote spectroscopic sensing owing principally to the laser pulse duration. We present a review of ultrafast laser-based spectroscopy techniques and discuss the use of these techniques to current and potential chemical and environmental sensing applications.

## 1. Introduction

While laser-based techniques have become a basic tool for remote and stand-off spectroscopy and sensing for environmental and security applications [1], the use of ultrafast lasers with pulse lengths in the tens of femtoseconds (fs) has, until recently, been limited to laboratory experiments [2]. The introduction of self mode-locking in Ti:sapphire based lasers in 1991 [3] brought simplicity of use and commercialization of ultrashort laser pulse technology so that 10 fs laser pulses can be routinely used in laser applications. In addition to the oscillator development, the development of diode-pumped solid state lasers as pump sources has helped to reduce the size of such laser systems. Chirped pulse amplification using solid-state gain media in regenerative or multipass schemes now routinely generates pulses with tens of femtoseconds and millijoule pulse energies at 1-kHz repetition rates or hundreds of millijoules at 10 Hz. The demonstration of femtosecond pulse generation in fiber lasers [4] and the follow-on development of ultrafast fiber laser technology has moved ultrafast laser applications such as THz time-domain spectroscopy from the optical table into hand-portable systems allowing for stand-off sensing in field applications.

Interest in ultrafast lasers for sensing arises primarily from their high time resolution at the timescale of chemical or electronic and vibrational processes and from the high peak power achieved in the pulses. Typically, these lasers operate in a very limited wavelength range, around 800 nm for Ti:sapphire or 1,550 nm for Erbium doped fiber lasers. The high peak power, though, allows conversion of the laser light to a wide range of different wavelengths. It makes non-linear frequency conversion very efficient, and multiple-harmonic generation has been used to generate coherent deep-UV and soft X-ray pulses [2]. Optical parametric amplification allows generation of femtosecond pulses at wavelengths in the near and mid infrared to 20 μm covering the fingerprint area of the IR spectrum which is especially useful for chemical sensing applications. To continue the spectral coverage, optical rectification allows generation of THz pulses from the mid-IR region (30 THz = 10 μm) to mm-waves at tens of GHz.

Beyond narrow bandwidth pulses, at high laser intensities the refractive index of any material, including air, becomes a function of the intensity, with the optical Kerr effect being the strongest, non-vanishing nonlinearity that occurs in all media. When properly controlled, this nonlinearity can give rise to a time-dependent phase shift and associated spectral broadening in the ultrashort pulse. This allows the generation of so-called white-light continuum pulses with significant powers and wavelengths ranging between 400 nm to 4.5 μm from a 800 nm fs pulse after traveling through short glass or sapphire fibers [5,6]. This type of source can be a valuable broadband ultrafast laser light source for sensing and spectroscopy applications.

Other nonlinear effects resulting from high power femtosecond laser interactions with air include filament generation [7,8], Raman signatures [9], laser-induced fluorescence [10] or stimulated backscattered emission [11,12] from these filaments that can also be used for sensing applications.

The applications discussed in depth in this article are Laser-induced breakdown spectroscopy (LIBS), coherent anti-Stokes Raman spectroscopy (CARS), and THz time-domain spectroscopy. For LIBS, the high power and short duration of the laser pulse shifts the emission spectra from atomic emissions to molecular emissions. The spectral width of an ultrashort laser allows CARS to be performed with a single laser, reducing complexity for many sensing applications. Finally, the high temporal resolution achievable using fs pulses allows the measurement of THz electric fields in the time-domain, generating an alternative tool to Fourier Transform Infrared Spectroscopy (FTIR) for far-infrared spectroscopy. Note that these and the techniques mentioned in the introduction do not complete the ever-growing list of possible effects and applications of ultrashort lasers for sensing and spectroscopy but provide a sampling of the various ultrashort-related effects that have been used to modify or extend existing approaches.

## 2. Laser-Induced Breakdown Spectroscopy

In this section we will focus on emission spectroscopy techniques that employ ultrafast, high-fluence, optical excitation. Among these, the technique that has been most widely investigated is laser-induced breakdown spectroscopy (LIBS), an analytical technique based on the spectral analysis of optical emission from laser-induced plasmas [13]. This technique has inherent stand-off detection capability, requires a very small amount of material for analysis and can perform at high detection rates making it attractive for analysis of organic and inorganic materials in a variety of circumstances [14,15]. It takes approximately one second or less to acquire a broadband spectrum for LIBS allowing for rapid materials analysis with the primary limitation being the read-out time of the spectrometer used to measure the emission spectrum [16].

Pulsed lasers can easily achieve the required conditions for LIBS materials analysis since the rates of energy deposition greatly exceed those of energy redistribution and dissipation with the result that extremely high temperatures can be achieved in regions where energy absorption occurs. Even so, the interactions of femtosecond laser pulses with materials are substantially different from those of nanosecond laser pulses since the rates of energy deposition are significantly higher. This leads to a range of material responses that ultimately affect LIBS measurements. For example, material removal [17,18] and plasma expansion characteristics [19] can vary significantly with excitation pulse duration. For materials with complicated chemical compositions, femtosecond excitation can yield ejecta resembling bulk stoichiometry [20,21] resulting in LIBS spectra that can be used to assess composition with tighter confidence intervals than for nanosecond excitation [22]. Also, ultrafast excitation results in a smaller heated volume around the ablation region generally leading to material removal processes that are more reproducible [23,24]. For molecular solids, ultrafast excitation has the potential to improve the analytical capability of LIBS, since mass spectrometry studies have shown high mass fragment and cluster formation under femtosecond laser irradiation [25] as well as optical emission from small molecules characteristic of the irradiated solid-phase species [26]. Optical emission obtained from molecules formed as a result of ultrafast excitation could improve the specificity of detection. For nanosecond LIBS, spectroscopic interpretation is based primarily on atomic emission [27]. This can be enhanced for both nanosecond and femtosecond ablation events through a variety of means including plasma plume excitation using a second laser pulse [14]. Enhanced emission from molecular species in dual-pulse experiments has also been reported for nanosecond ablation events and has been used for identification of molecular overlayers [28]. In this section, we will highlight differences between single pulse nanosecond and femtosecond laser-induced breakdown spectroscopy of relatively thin, organic molecular overlayers residing on surfaces for the purpose of sensing of these molecular layers [29,30].

It is widely recognized that there are gaps in our ability to model various processes that can occur during ultrafast excitation of materials surfaces–especially those that are composed of organic species on substrates. First of all, the electromagnetic and transport properties of electron and phonon systems over a wide range of temperatures and pressures are not well-understood. Existing models use continuum approaches and employ properties such as the optical absorption depth, the electron and phonon thermal conductivities, the electron and phonon heat capacities and the electron-phonon coupling parameter. These properties are generally known for a limited number of materials under a restricted range of conditions. Stress field development at short times can result in a variety of behaviors including cavitation of molten material and spallation of solid phase material. Continuum models can be employed effectively in predicting the onset of these behaviors but can be problematic in attempts to model system evolution beyond onset. Material ablation is poorly understood since many materials states/phases can coexist in a single event and evolve rapidly in time. Consequently, there is no preferred modeling approach that can adequately describe the material behavior for an entire ultrafast laser excitation event. Molecular dynamics simulations have the ability to fill in critical gaps if interatomic potential functions are used that are appropriate to the prevailing conditions near ablation. Since LIBS sensing requires detection of emission from species present during the ablation event, spatio-temporal plume energy evolution plays an important role in determining characteristic signals. For nanosecond laser pulses, photons arriving later in the pulse can interact with material residing in the plume itself–the laser pulse essentially interacts with a range of states and will couple strongly to those that are available and accessible. In contrast, femtosecond laser pulses primarily interact with initial electronic states of the material through single photon or multiphoton processes. Consequently, femtosecond pulses produce a more restricted and potentially predictable cascade of energy states since additional photons are not delivered at timescales during which ablation occurs. Even so, there can be a range of interactions among excited state species after the initial irradiation event – these might or might not be useful for sensing purposes.

**Figure 1.**
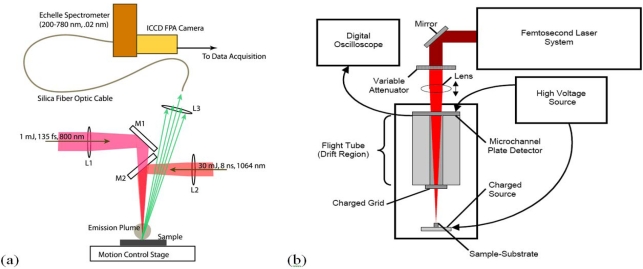
**S**chematic diagrams of the experimental apparatus used to collect **(a)** laser-induced breakdown spectroscopy (LIBS) signals and **(b)** femtosecond time-of-flight mass spectra.

A LIBS system schematic is shown in Figure 1 that illustrates excitation with either nanosecond or femtosecond laser pulses. Most femtosecond LIBS studies to date have been performed using Ti:Sapphire based laser systems. Readily available commercial systems can produce pulses with durations in the 30–150 fs range with energies of 1–100 mJ at repetition rates up to 1 kHz. The wavelengths for excitation are restricted primarily to those that can be developed with sufficient energy to produce emission. Typically, studies have been performed with wavelengths around 800 nm or at wavelengths corresponding to frequency doubled or tripled radiation. In laboratory studies, laser outputs are focused with lenses to produce the fluences/fluxes required for plasma formation. The optical emission from the plasma is collected using standard reflective or refractive optics and is analyzed and recorded using spectrometers optimized for transient optical signals. For results shown here, an Echelle-type spectrometer was used (200–785 nm, resolution of 0.02 nm) with a computer interfaced intensified charge-coupled device (ICCD) camera. The image intensifier in the camera was controlled using gating electronics and a digital delay generator internal to the camera head provided gating of the recorded spectrum with an adjustable delay and width. LIBS events and emission spectrum collection were synchronized using delay generator electronics. To assist with the interpretation of LIBS spectra, it is often useful to measure other ablation plume characteristics especially when the interaction is poorly understood. In our work, we have measured ionized products using a time-of-flight mass spectrometer since this permits identification of molecular species produced when organic materials are excited. A schematic of a single femtosecond pulse, ablation-ionization, mass spectrometer system is also shown in Figure 1.

**Figure 2.**
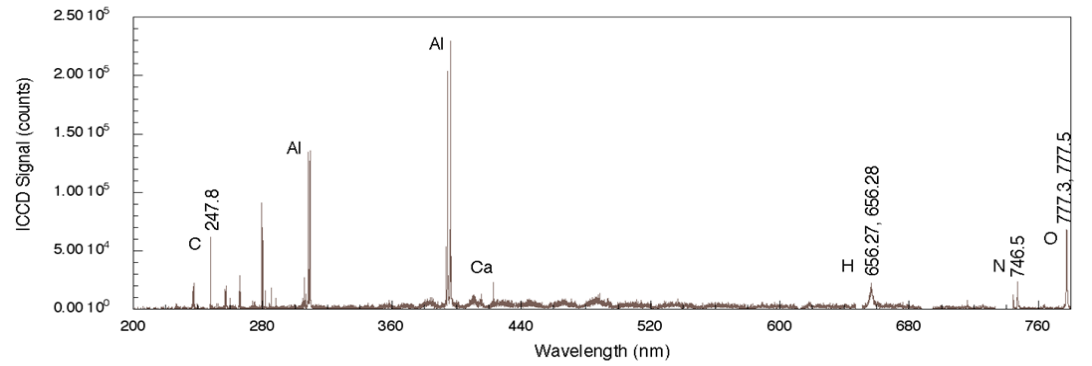
Nanosecond LIBS signal obtained from TNT on an aluminum substrate. Prominent emission lines from the substrate are present along with lines associated with TNT (C, H, N, O) and contaminants (Ca).

To illustrate the general characteristics of femtosecond LIBS for sensing of organics on surfaces, we will focus on simple compounds used in explosives since these have been studied extensively in the literature. Nanosecond LIBS spectra for a number of explosives including TNT, RDX, HMX, and PETN has been reported previously and these generally highlight emission from the constituent elements of nitrogen-bearing explosives (C, H, N, and O) [31]. Stand-off detection of explosives has also been reported using molecular emission from C_2_ and CN in addition to the elemental emission for detection and identification [32]. Figure 2 shows a nanosecond LIBS spectrum for TNT on aluminum with single-pulse excitation [29]. The aluminum substrate strongly reflects the incident radiation but absorbs sufficient energy to allow for plasma formation. While it is not clear whether the explosive is directly excited by the laser pulse owing to nonlinear processes or by heating from substrate-dominated processes, emission from the elemental constituents of the explosive is observed. The four strongest emission lines in the spectrum are associated with the aluminum substrate and occur at 308.22, 309.28, 394.42, and 396.16 nm. The relative intensities of these lines relate to various characteristics of the plasma including its temperature as well as the number density of emitting species but also depends on the design of the emission collection system [33]. Emission associated with atomic species from the explosive occurs at 247.86 nm (carbon), 656.56 nm (hydrogen), 747.02 nm (nitrogen), and 777.32 and 777.52 nm (oxygen). The oxygen-related emission is the result of three closely-spaced transitions for neutral oxygen (777.19, 777.42, and 777.54 nm) [34]. Additional peaks present in the spectrum, such as the one related to Ca, are most likely related to surface contamination of the substrate before deposition of the explosive solution. The results reported here throughout the 200–800 nm region clearly show significant substrate-related emission. In addition, owing to the duration of the laser pulse, there can be significant excitation of environmental species above the absorbing substrate and these can contribute to the recorded spectrum but the extent of this contribution is not known for the results shown here. In addition, chemical reactions in the laser-generated plasma between the excited solid and environmental species can also generate emission indirectly related to the solid [35]. Even so, by employing algorithms assessing various line intensity ratios, explosives can be discriminated from other samples and the effects of atmospheric species can be taken into account [30].

**Figure 3.**
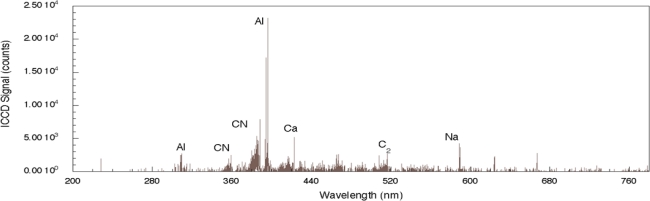
Femtosecond LIBS signal obtained from TNT on an aluminum substrate over the complete detection wavelength range. Elemental emission from the substrate and contaminants are present along with emission from CN and C_2_.

**Figure 4.**
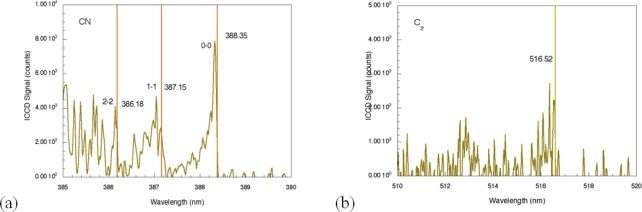
Higher resolution spectra of selected regions of the spectrum obtained for femtosecond excitation of TNT on aluminum. **(a)** Expanded view of emission from CN. **(b)** Expanded view of emission from C_2_. The detection gate delay and width were 100 ns and 1 μs, respectively.

For comparison, the femtosecond LIBS signal for TNT on aluminum is shown in Figure 3 [29]. For this spectrum, the acquisition gate delay and width were shorter than for nanosecond spectrum acquisition since background continuum emission from femtosecond plasmas decays more rapidly than for nanosecond excitation [23,24]. In addition, the duration of elemental emission is shorter requiring spectrum acquisition closer to the time of surface irradiation [24]. As was the case for nanosecond LIBS of TNT on aluminum, the two strongest emission lines are associated with the aluminum substrate (394.42 nm and 396.16 nm). The other aluminum emission lines previously noted (308.22 and 309.28 nm) are also present, but the relative intensities of these lines compared to the strongest aluminum emission lines are smaller than occurs for the nanosecond case [29]. The most significant differences between the nanosecond and femotsecond spectra are the absence of atomic emission in the femtosecond spectrum for species associated with TNT along with the presence of molecular emission that can be attributed to CN and C_2_–these are highlighted in Figure 4. The CN emission (370–400 nm) is the result of sequential electronic transitions from the second excited to the ground electronic level with no change in the vibrational quantum number [36]. Transitions beginning in different vibrational levels of the excited electronic level lead to characteristic spectral emission bands with the strongest being related to the (0, 0) band (388.32 nm). Others are labeled in Figure 4 as well [29]. The CN vibrational temperature determines the relative peak amplitudes of these emission bands while the rotational temperature contributes to the shape of a particular vibrational band. This is observed most clearly for the (0, 0) band of the CN spectrum. Initial attempts to model a related femtosecond-excited, CN emission spectrum using LIFBASE simulations [37] yielded different values for the rotational and vibrational temperatures–5,000 and 12,000 K respectively. The origin of this discrepancy is not fully understood at this time since related simulations for nanosecond pulse excitation of systems producing CN emission yield equal rotational and vibrational temperatures and can be used to identify molecule formation processes [35]. The C_2_ spectral emission (highest intensity at 516.54 nm) is also composed of electronic transitions with no change in the vibrational quantum number but these have not been modeled for the data shown here. The absence of atomic emission associated with the explosive in the case of femtosecond excitation [38] is most likely a result of the particular excitation conditions (pulse fluence) used to obtain the results presented here. Recent reports of fluence dependence [26] indicate that the ratio of atomic-to-molecular emission increases as femtosecond pulse fluence is increased. The origin of the molecular species is either fragment production during the initial excitation event or fast chemical reactions among atomic species generated during molecular breakdown. In either case, it appears that higher fluences decreases the production/proportion of excited state molecular species that contribute to femtosecond LIBS signals.

**Figure 5.**
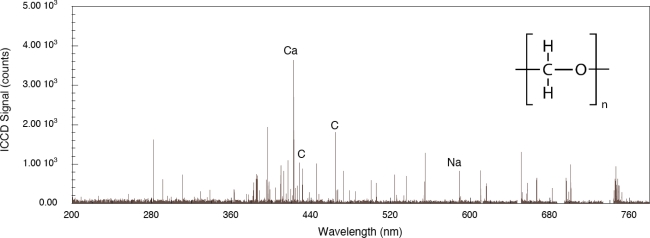
Femtosecond LIBS spectrum of polyoxymethylene (Delrin^®^) loaded with a carbon filler.

**Figure 6.**
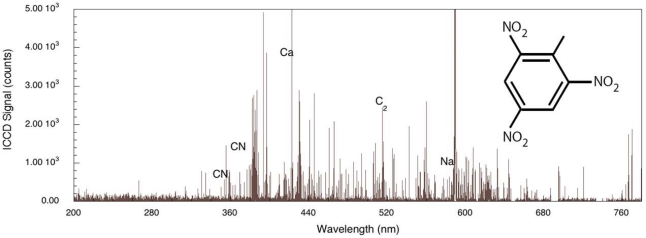
Femtosecond LIBS spectrum of TNT on polyoxymethylene with a carbon filler.

**Figure 7.**
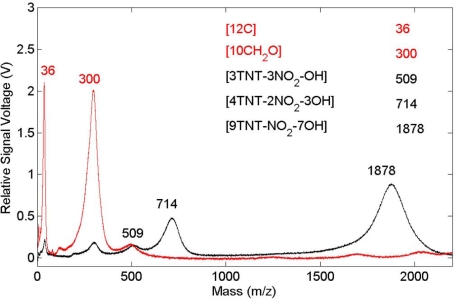
Femtosecond laser excited time-of-flight mass spectrum of polyoxymethylene (light) and of TNT on polyoxymethylene (dark).

The role played by the substrate in the interaction is important for both femtosecond and nanosecond excitation. For the system considered here, a thin layer of explosive on a substrate, the explosive has weak linear absorption for the laser wavelengths used and a significant portion of the laser energy is absorbed by the substrate. Energy is transferred to the layer either directly by excited electrons, indirectly by phonons or through other types of collisions. The first two of these processes have been described previously for simple adsorbate-substrate systems subjected to relatively low pulse fluences [39]. The effect of organic substrate systems are particularly interesting since these provide wholly different excitation pathways leading up to LIBS signal generation. To demonstrate organic substrate-related effects, a carbon-loaded polyoxymethylene (Delrin^®^) material has been used to generate the femtosecond LIBS spectrum shown in Figure 5. Compared to the aluminum system, LIBS signal intensities are lower owing to decreased evolution of electronically stimulated processes – optical absorption in the polymer is not localized to the material surface through interaction with conduction electrons as occurs in metals. Atomic emission from calcium, carbon and sodium are observed but there appears to be no molecular emission or emission from oxygen or hydrogen which are significant components of the polymer. This indicates that carbon-related emission is from filler material compounded into the polymer and that the polymer itself is excited in ways that do not result in emission in this spectral range. The corresponding femtosecond LIBS spectrum of TNT on polyoxymethylene is shown in Figure 6. The carbon emission lines present in the polyoxymethylene spectrum are essentially missing in this spectrum but CN emission occurs indicating that, once again, the presence of TNT on the surface results in CN formation and emission. These results show that CN is derived primarily from the TNT molecule and is not associated with reactions involving environmental species. Related time-of-flight mass spectrometry results for polyoxymethylene and TNT on polyoxymethylene, shown in Figure 7, indicate that fragments of the polymer itself are removed from the surface primarily as clusters.

Under the excitation conditions used for the results presented here, it is clear that molecular emission processes are more important for femtosecond LIBS than for corresponding nanosecond LIBS measurements. Femtosecond LIBS spectra as well as femtosecond TOF mass spectra show important differences between the interactions of metallic and organic substrates with organic overlayers. At this time, a better understanding of the respective signal generation processes is needed to exploit the fundamental differences between the two excitation regimes in a variety of materials systems.

## 3. Single-Beam Coherent Anti-Stokes Raman Scattering Spectroscopy Using Ultrafast Lasers

**Figure 8.**
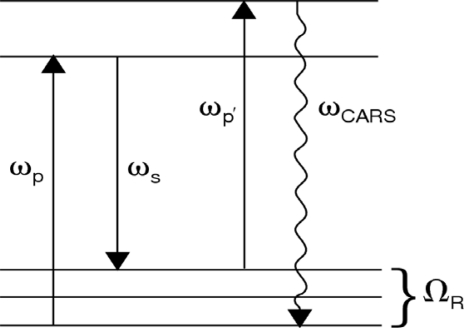
Schematic diagram depicting coherent anti-Stokes Raman scattering. ω_p_, ω_S_, and ω_p’_ represent the pump, Stokes, and probe sources, respectively. Ω_R_ is the vibrational excitation of the medium that is coherently excited by the pump and Stokes sources. Scattering of the probe source by the vibrational excitation results in the anti-Stokes beam at frequency ω_CARS_.

As a molecular spectroscopic tool, Raman scattering is one of the most powerful and widely utilized methods for the identification of molecules and the characterization of their properties. With the development of high peak-power, pulsed laser sources, a host of coherent Raman techniques have been developed for materials characterization and analysis (see, for example, [40,41]). In this section, we address coherent anti-Stokes Raman scattering spectroscopy, termed “CARS”, using a single ultrafast laser for excitation. In general, CARS is the most commonly employed nonlinear optical spectroscopic tool for the characterization of molecular structure and dynamics. Similar to spontaneous Raman scattering, the CARS process can yield chemically relevant information associated with the photo-excited medium via the molecular vibrational signature that is present in the scattered light signal. In this nonlinear optical process, shown schematically in Figure 8, a pump photon, ω_p_, a Stokes photon, ω_s_, and a probe photon, ω_p’_, mix coherently to emit a signal photon at frequency ω_CARS_. The energy level diagram in Figure 8 shows that resonant enhancement occurs when the energy difference between the pump and Stokes photon coincides with a vibrational level of the medium [42,43]. In addition to energy level considerations, efficient coupling between the incident laser and the anti-Stokes fields requires momentum conservation, *i.e.*, phase-matching, which generates a highly directional and intense CARS signal. In many instances, the coherent nature of the nonlinear interaction can provide signal enhancement factors on the order of 10^9^ with respect to the spontaneous phenomena.

From a fundamental point of view, the total Raman response from an illuminated medium arises from two underlying molecular mechanisms. In the spontaneous Raman mechanism, inelastic scattering of photons from molecular vibrations can produce both Stokes and anti-Stokes radiation. The Raman emission is incoherent, which results in an intensity profile that has no characteristic peak in the forward scattered direction. The emission intensity for both the Stokes and anti-Stokes interactions are quite weak, typically six or more orders of magnitude below the excitation source intensity [44]. This characteristic tends to limit applications associated with stand-off detection of chemical materials to distances on the order of 10 to 100 meters [1].

The second fundamental Raman scattering mechanism arises when optical sources of sufficient intensity are used for the materials excitation. Under these conditions, the pump and Stokes fields (ω_p_ and ω_s_ in Figure 8) can generate a vibrational excitation (Ω_R_) that coherently oscillates at the difference frequency of the two laser fields. The excitation can couple through a third-order nonlinearity with the probe field to produce an anti-Stokes output [40]. When cw and longer pulsed lasers (nanosecond to picoseconds) are used for the excitation sources for CARS generation, their narrow spectral bandwidth requires two individual laser systems for experimental applications. More importantly, one of these sources must be tunable if spectroscopic information is to be gathered from the measurement, which places a limitation on the ability to rapidly generate a CARS spectrum from the excited medium.

Although considerable materials characterization has been demonstrated with conventional CARS spectroscopy, the technique continues to have widespread utilization within many researcher and development communities. In part, this activity is being driven by the availability and relative affordability of high peak-power femtosecond lasers. From an emerging applications point of view, fs CARS spectroscopy offers considerable potential for the challenging problems associated with standoff detection of chemical and biological materials. These two factors have been paramount in the continued transition of conventional CARS to the world of ultrafast laser systems, since the temporal and broad spectral characteristics of many fs laser systems enable the discovery of novel material signatures that can be utilized for future applications such as chemical detection.

With the emergence of fs CARS, we focus our attention on an approach that is expected to have a significant impact on applications associated with standoff chemical detection, namely, fs CARS using a single-beam source [45]. This arrangement has been utilized by numerous groups for CARS analysis, where a single fixed-frequency, ultrafast fs laser is used as the broadband pump, Stokes, and probe sources in Figure 8. In single-beam, fs CARS, a coherent molecular vibrational mode in the illuminated medium is generated by the broadband pump and Stokes lasers, which is then probed by an additional broad probe pulse. To date, most fs CARS measurements have utilized Ti:sapphire-based lasers, which provide a source of pulse widths and peak powers that can generate easily detectable CARS signals from gas, liquid, and solid samples. The high peak power of many commercially-available fs lasers provide efficient excitation of two-photon Raman transitions by providing a resonant photon pairs, *i.e.*, pump and Stokes sources, within their wide spectral bandwidths for the coherent excitation of any accessible transition [11,46,47]. It is noted that CARS investigations with multiple fs lasers is also an exciting area of nonlinear spectroscopy, but this topic will not be addressed in this review. Readers are encouraged to examine a number of excellent examples of chemical detection and analysis with multi-beam fs CARS in the following references [47–50].

Although a single-beam approach reduces the experimental complexity associated with conventional CARS optical sources, the broadband nature of the fs source produces two challenges that must be addressed prior to spectroscopic analysis. First, the resolution of the vibrational features within a CARS spectrum are directly related to the bandwidth of the ultrafast laser source, which exceeds hundreds of wavenumbers for pulses on the order of 20 to 30 fs [51]. Without corrective measures, this characteristic imposes poor spectral resolution on the vibrational features within the CARS spectrum when compared to corresponding features in spontaneous Raman emission. The second concern is associated with the large non-resonant CARS component (*i.e.*, relative to the corresponding term that is generated in a narrowband CARS measurement), which can overwhelm the contribution from the vibrational resonant component. Over the past decade, these two issues have been successfully addressed with a host of coherent control approaches that rely on conditioning the phase and/or polarization of the incident fs source. The use of coherent control has been shown to enable the generation of CARS spectra that span a considerable region of the molecular vibrational region (200 to 3,000 cm^−1^) with high resolution (∼30 cm^−1^) and minimal non-resonant background [51–56]. In addition to the enabled spectroscopic capability, some rather creative phase and polarization algorithms have been developed that control the coupling of the pump source with specific vibrational modes in an illuminated medium, which provides a new tool for selective detection of a relevant analyte within a chemically complex sample [57].

**Figure 9.**
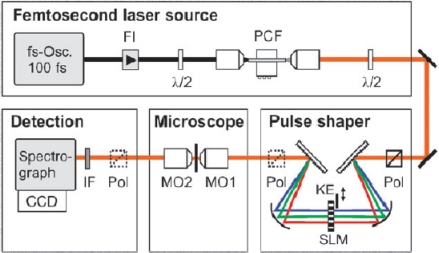
Experimental setup for a fs CARS measurement [57]. Details of the arrangement are discussed in the text. Briefly, broadband fs pulses are created with a standard 100 fs oscillator and broadened into a supercontinuum using a photonic crystal fiber (PCF). The pulse shaper for the CARS measurements consists of a pair of gratings, a liquid crystal spatial light modulator (SLM) and spherical mirrors. Additional system details can be found in [57]. Reprinted from von Vacano *et al.* [57]. Copyright 2007, American Institute of Physics.

A fs CARS experimental system is shown in Figure 9, which illustrates the respective optical components that enable both phase and polarization shaping of the fs pulse for this nonlinear spectroscopic technique [57]. The source laser is a standard 100 fs Ti:sapphire oscillator, which is coupled to a photonic crystal fiber (PCF) to increase the bandwidth of the incident radiation via supercontinuum generation [58]. The PCF enables the generation of a supercontinuum spectrum that has a pulsewidth on the order of 15 fs. Following the generation of the broadband source, this pulse is then “conditioned” for fs CARS by a combination of free-space optics and spatial light modulators (SLM). First, the fs pulse is transformed from the time domain into the frequency domain by a dispersion grating prior to the SLM, where the spectral profile of the pulse is linearly dispersed along the plane of the device. Due to the pixilated nature of the SLM, each array element can impart a pre-programmed phase and polarization value to the spectral component that is transmitted through that specific region of the device. Typically, SLMs have hundreds of individual pixels, which enable the construction of a phase and polarization profile on the fs pulse with ∼0.5 nm resolution. After SLM transmission, the pulse is transformed back into the time domain with a second dispersion grating, where the temporal field profile of the conditioned pulse is essentially the Fourier transform of the SLM-imposed profile on the electric-field spectrum.

For the setup in Figure 9, the SLM has been programmed to produce both the broad band pump and narrow band probe beams for fs CARS. In regards to polarization control, the SLM device rotates the electric field of a narrow band of the fs pulse spectrum into a direction that it is orthogonal to the remainder of the pulse. Under these polarization conditions, the CARS signal that arises in the plane of the probe beam will depend on the product of the probe and the driving polarization field that arises from a second-order nonlinearity within the illuminated material. Since the electric-field of the probe pulse is zero outside the spectrally narrow region, the bandwidth of this CARS component is determined by the probe itself, *i.e.*, the limiting resolution of the SLM device. In dealing with the reduction of the nonresonant CARS background, the investigators program the SLM to split the narrow band probe beam into two distinct sources by imposing a π phase step at the center of the probe frequency. Upon mixing this two probe sources with the pump beam, the two nonresonant CARS signals that are generated are of equal amplitude but opposite phase, which leads to a near zero nonresonant response from the illuminated material.

**Figure 10.**
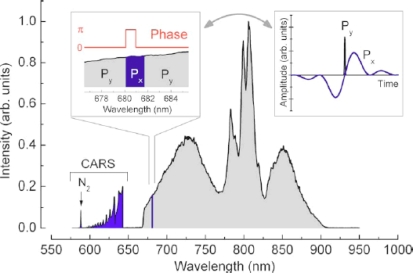
Spectrum of the 7 fs laser beam and detected CARS signal from a measurement reported in [59]. In the left inset, the phase and polarization masks are shown; *Px* and *Py* correspond to orthogonal polarizations. In the right inset, the temporal profiles of the excitation and probe parts of the beam are shown along with their overlap. Reprinted from Roy *et al.* [59]. Copyright 2009, American Institute of Physics.

This form of pulse shaping approach was introduced by Oron *et al.* [51] and has been shown by other investigation to address both resolution and background issues associated with single-pulse, fs CARS. Roy *et al.* [59] have recently utilized this same conditioning algorithm for a spectrally broadened fs pulse, which is shown in Figure 10. The data in this figure illustrate the phase and polarization distribution (left inset) that is imposed on the fs spectrum pulse via the pulse shaping optics that are used for beam conditioning. The conditioning resulted in a spectral resolution of ∼10 cm^−1^ within the CARS spectrum, which allowed the investigators to probe the effects of pressure and mixture concentration on the stretching frequency of molecular nitrogen.

In Figure 9, it is also noted that an additional “conditioning” step must be performed on the fs pulse prior to sample excitation. Since the incident pulse is sufficiently broadband to include wavelengths within the CARS spectral region, it is critical to eliminate this component prior to sample illumination since it will interfere with the detection of the CARS signal via the spectrograph and detector array. This can be easily accomplished by placing a knife-edge (KE) within the pulse shaper, where the short-wavelength region of the supercontinuum is physically blocked by the sharp aperture. Finally, although CARS detection is accomplished with conventional spectroscopic hardware (e.g., spectrograph and array detectors), it may be important to incorporate other “conditioning” optics such as polarizers in the incident and detection pathways. These components are typically employed to enable the highest resolution and nonresonant rejection in CARS spectra.

**Figure 11.**
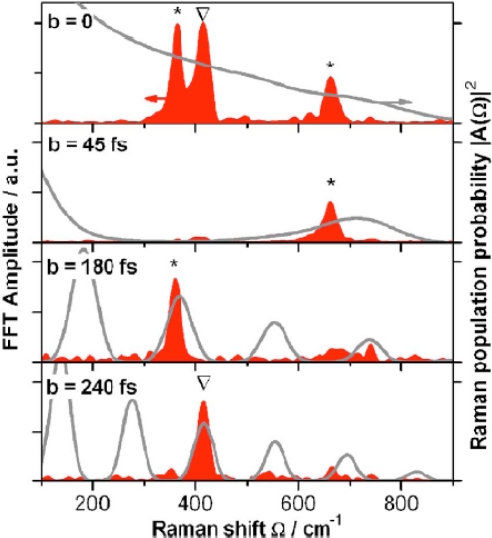
fs CARS spectra from [57]. Control results for molecular vibrations selectively excited in a binary mixture of CHCl_3_ and CBrCl_3_ with time-resolved single beam CARS. A suitable multipulse spacing *b* achieves full selectivity and control over all the accessible modes of CHBr_3_ (marked “Δ”) and CHCl_3_ (marked “*”). Reprinted from von Vacano *et al.* [57]. Copyright 2007, American Institute of Physics.

As an illustration of the general characteristics of fs-based CARS for chemical analysis, we will consider two examples that highlight the unique features that are afforded this technique as a result of pulse shaping. It is difficult to overstate the impact that this variable imparts to fs CARS, since the large freedom associated with coherent control of the excitation profile will likely impact the coupling, both spectrally and temporally, to the illuminated media. In the first illustration, which uses the experimental setup shown in Figure 9, von Vacano *et al.* demonstrated the ability of a coherently controlled fs pulse to selective couple to specific vibrational modes within a complex organic liquid mixture [57]. An example of the selectivity afforded via coherent control is illustrated in Figure 11, which show CARS spectra from a 5:1 mixture of CHCl_3_ and CBrCl_3_. The pump and probe beams for this measurement are generated from a broadband fs pulse that is processed with a SLM device to generate an output profile that has a first pulse that coherently excites a Raman vibration in the molecular sample and is subsequently probed with a delayed second pulse. The delay between the pump and probe beams is variable, which enables an investigation of how this pulse delay affects the selective excitation of a molecular mixture. As is shown in Figure 11, the ability to vary the temporal delay between the excitation pulses has a pronounced effect on molecular coupling. In this figure, the spectra from top to bottom show a progressive change in the ability to excite vibrational modes within this binary mixture. Through coherent control, the investigators were able to construct an excitation profile that was comprised of a series of pulses that were separated by *b* = *nT*_vib_, with *n* being an integer number and *T*_vib_ the respective vibrational period. By varying the delay through pulse shaping, the CARS response shows that it is possible to selectively excite a single molecular vibration within the solvent mixture. With little or no delay (top spectrum), there is clear indication of exciting both the CHCl_3_ and CBrCl_3_ vibrational modes in the solution, which is similar to results obtained with spontaneous Raman scattering. However, as the delay is increased, it is possible to selectively excite individual vibrational modes in the binary mixture. In the bottom spectrum, the investigators demonstrate the ability to eliminate coupling to the dominate component in the mixture, which highlights the potential of this technique for highly selective detection of chemical analytes at low relative concentrations.

**Figure 12.**
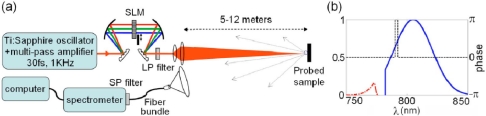
(a) The experimental setup, and (b) an illustration of the spectral amplitude (blue) and phase (dashed black) of the phase-gated laser pulse employed for femtosecond CARS in [60]. Details of the setup and spectral source are in the text. Reprinted from Katz *et al.* [60]. Copyright 2008, American Institute of Physics.

**Figure 13.**
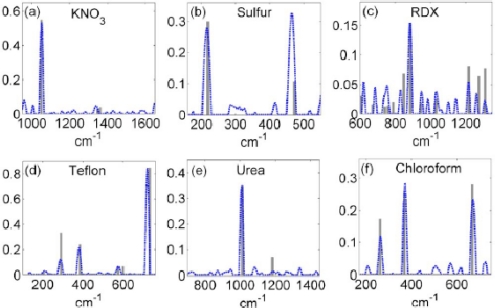
CARS spectra from [60]. Resolved femtosecond CARS vibrational spectra of several scattering samples (dashed blue), obtained at standoff distances of [a–c] 12 m and [d–f] 5 m (a) 1,000 μg crystallized KNO_3_, (b) <500 μg sulfur powder, (c) Cyclotrimethylene-trinitramine (RDX/T4) explosive particles with a total mass of <4 mg, (d) bulk PTFE, (e) <4 mg of crystallized urea particles, and (f) 1 cm long cuvette containing chloroform and scattering ZnTe particles (200 nm diameter). Additional details of the experimental measurement for this data can be found in [60]. Reprinted from Katz *et al.* [60]. Copyright 2008, American Institute of Physics.

In addition to the chemical analysis within complex media, fs CARS can also be employed for application that require standoff detection motifs. An example of a recent the CARS measurement designed for standoff detection is shown in Figure 12 [60]. In this investigation, Katz *et al.* employed a spatial light modulator for pulse shaping of the fs pulse, which is analogous to the previous example. An illustration of their setup and spectral profile of their excitation source is shown in Figure 12. For the standoff measurements, the conditioned fs beam was focused on a distant sample (5 to 12 meters) and scattered radiation was collected with standard telescopic lens in a backscattered geometry. Due to the pulse shaping within the SLM, a narrow-band probe was defined within the excitation spectrum by shifting the phase by π in a narrow frequency range (dashed line in Figure 12b). The phase-shifted probe beam allowed the resonant and nonresonant CARS signals to interfere constructively at the high frequency side of the gate, generating a peak in the CARS spectrum, and destructively at the low frequency side, generating a dip in the CARS spectrum. In these results, the authors achieved high-resolution standoff capability by subtracting the CARS spectra with a transform limited, flat-phase pulse that was obtained with the π phase-gated pulse. With this approach, the vibrational Raman spectrum was extracted from the as-collected data, which highlight the resolved vibrational spectra of trace levels of solids, liquids, and explosives particles that were examined in the investigation. These latter results are shown in Figure 13.

Overall, the advances in single-pulse, fs-CARS experimental systems for both laboratory and field-related chemical detection continue to demonstrate the potential of this technique for real-world chemical analysis and detection. The development of ultrafast CARS affords a new toolbox for analysis and detection. In addition to the benefits associated with large spectral bandwidth, the ability to coherently control the excitation profile of the fs source enables a broad range of CARS applications that require selective detection within complex environments. Finally, the ultrafast source will likely enable a new set of applications associated with probing material dynamics or modification their chemical properties. Among applications that may overlap with future CARS measurements, phase-shaped pulses will likely play a role in controlling multiphoton absorption [61], chemical reactions [62], and high-harmonic generation [63].

## 4. THz Spectroscopy

Terahertz (THz) spectroscopy is a technique that exploits low energy modes of materials, where all the atoms within the molecules participate in collective motions yielding unique spectral signatures of many organic molecules in the terahertz frequency range. Some phonon modes can also be seen in the terahertz frequency range in solid materials. The collective motions of molecules in this frequency band allow spectral fingerprinting, different from what is seen in the infrared, which lends itself to unique identification of materials by terahertz spectroscopy. For solid materials, these collective motions not only include molecular motions involving all the atoms within one molecule (intramolecular motions), but also collective motions of multiple molecules within the sample (intermolecular motions). Terahertz radiation is also highly sensitive to polar molecules like polar liquids and gases. Many polar gases exhibit unique spectral signatures from probing transitions between rotational quantum levels [64] while probing polar liquids allows low-frequency intermolecular spectroscopic information to be obtained [65].

THz time-domain spectroscopy (TDS) benefits from the broad bandwidth available through the use of femtosecond lasers as well as the high signal-to-noise that is achievable. Bandwidths up to near 40 THz have been shown [66] through the use of femtosecond laser techniques for THz generation and detection. The ability to generate broad bandwidth signals in combination with high signal-to-noise, THz TDS has the ability to probe many condensed-phase systems that unique spectral characteristics in this portion of the spectrum. THz TDS, being a coherent detection technique, also has the ability to probe gaseous systems, like performing the spectroscopy of flames whereas traditional Fourier Transform Infrared (FTIR) techniques does not allow for spectroscopy of hot samples like flames due to the sensitivity to incoherent radiation. THz TDS additionally benefits from direct measurement of the THz electric fields, which allows for direct measurement of the amplitude and phase information, where traditional Fourier Transform techniques only allow for measurement of the intensity, eliminating the opportunity to directly measure the phase information.

Terahertz technology has experienced strong growth [67–69] since the early 1980s when the field was able to benefit from advances in ultrafast laser techniques. Several techniques can be employed to generate and detect terahertz radiation with femtosecond lasers. Among the options for generation of THz radiation are electro-optic generation, photoconductive generation, and generation in air. Among the options for detection using femtosecond laser techniques are electro-optic detection, photoconductive detection and air detection. The variety of techniques can be used in combination with each other, e.g., one can use photoconductive generation techniques and electro-optic detection techniques together.

Electro-optic (EO) THz generation is a non-linear optical effect which exploits difference frequency mixing or optical rectification in electro-optic crystals. The broad spectral bandwidth of the femtosecond pulse allows the mixing of different spectral components through nonlinear (χ_2_) processes in the electro-optic material. This leads to generation of radiation in the terahertz frequency range for a variety of materials. Some materials which radiate in the terahertz frequency region are ZnTe, GaP, LiNbO_3_, and nonlinear optical polymers [70–74].

Photoconductive generation through photoconductive switching with femtosecond lasers exploits the direct bandgap of the material used for generation. The femtosecond laser excites electrons and holes in the semiconductor in the area between the electrodes (antenna structure). With a bias voltage applied across the electrodes, the electrons accelerate, generating terahertz radiation in the far-field. One material most commonly used for photoconductive THz generation is GaAs [75–78].

Generation of THz radiation in air has also been explored. Mixing both the laser fundamental (e.g., 800 nm) and the second harmonic (e.g., 400 nm) waves can yield THz radiation [79–82]. This process was initially attributed to four-wave difference frequency mixing in air through third-order nonlinear optical effects (χ_3_) [80,81]. Kress and co-workers [79] have shown that plasma generation is essential to air-generation of THz radiation as the nonlinear coefficient in air is insufficient to explain the resulting THz field strengths observed in the air-generation process. In addition, Kim and co-workers [82] have recently developed a transient photocurrent model to explain the coherent THz emission from air. This phenomenon can be exploited to enable remote generation of THz radiation for stand-off applications where it is not practical to propagate THz radiation large distances through the atmosphere.

**Figure 14.**
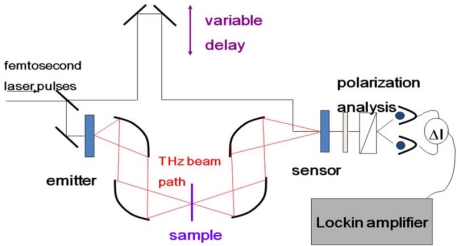
Sample THz time-domain spectroscopy experimental set-up.

Femtosecond detection mechanisms employ a pump-probe technique where a portion of the fs laser beam is split off for detection while the majority of the beam is used for generation of the THz signal (see Figure 14 below for a typical experimental set-up). The use of femtosecond lasers for detection of coherent THz radiation allows for the measurement of the electric field instead allowing for direct measurement of both amplitude and phase information. Other techniques commonly used in the THz frequency region are Fourier transform techniques where only the amplitude of the intensity is measured. Through the use of complicated Kramers-Kronig relations, amplitude and phase information can be calculated, however, the direct measurements provide the amplitude and phase information more readily and directly.

In electro-optic detection techniques, the THz electric field acts as an external electric field modulating the index of refraction of a nonlinear crystal where the probe laser beam experiences the electro-optic effect which can be directly measured at the laser wavelength with standard photodiodes. The change in the index of refraction of the material when the THz electric field is present modifies the polarization of the probe (detection) laser beam which can be directly measured using balanced detection techniques.

In photoconductive detection techniques, the THz electric field induces a current in the material which is directly measured across the patterned antenna structure. This process is the opposite of that occurring in photoconductive generation using a photoconductive switch or antenna structure. Similarly to electro-optic detection, air detection mixes the THz and laser beams resulting in a measurable signal with standard photodiode detection techniques.

**Figure 15.**
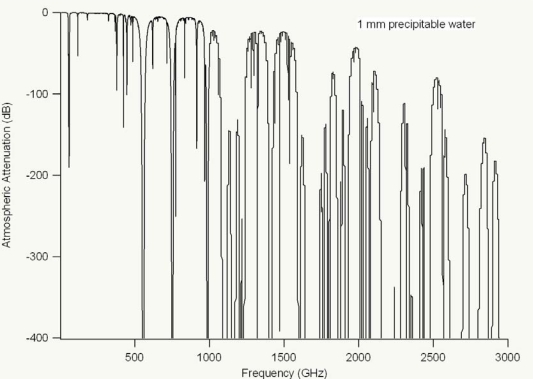
Atmospheric attenuation (in dB) of 1 mm precipitable water from 10 GHz to 3 THz. (Model calculations based on [102] www.smm.caltech.edu/cso/weather/atplot.shtml).

Limitations due to atmospheric attenuation (see Figure 15 below) prevent long-distance standoff spectroscopy techniques, although detection of explosives has been demonstrated up to 30 meters away [83]. Some of the main limitations for long-distance standoff applications, however, relate back to the available THz power generated by the emitter and the sensitivity of the detector. Increased power output and increased detection sensitivity, which will come with improvements to technology, will aid the ability of THz technology to overcome long-distance standoff technical hurdles. While there has been a multitude of research on THz spectroscopy of explosives [84–92], there has also been much interest in terahertz for other security applications [93–95] as well as in the pharmaceutical industry [96]. A variety of research also focuses on medical applications of terahertz spectroscopy and spectroscopic imaging including detection of skin cancer [97,98] and dental caries [99]. Since attenuation of terahertz radiation by water vapor is not a problem in outer space, there is considerable interest in terahertz for space-based applications including looking for gases and water on Venus, Mars, Saturn, and Titan [100,101]. Although many space-based techniques tend to use heterodyne techniques, as optical techniques improve, or become more readily located in observatories above the bulk of the atmosphere (e.g., at observatories in the Andes mountains in Chile or on top of Mauna Loa in Hawaii), ultrafast laser-based space sensing will continue to be used for space exploration.

The detection of gas-phase samples has been a major application of Terahertz Time-Domain Spectroscopy (THz TDS) and plays a role in many varying fields. Work has been done to investigate the absorption and dispersion of ammonia vapor (NH_3_)_2_ [103]. Further specification of gases, such as the free electron density and electron scattering time for ionized oxygen and nitrogen [104], has been achieved using THz TDS. Even the detection of trace gas samples, in the tens of ppm, has been demonstrated using this method [105]. Due to the sensitivity allowed by ultrafast pulsed lasers, differentiating between similar gas species in a mixture is quite possible. This has been realized for distinguishing between CH_3_CN and CD_3_CN [64] and separately for NH_3_ and H_2_O [106].

Extensive work has been done in the area of explosive detection using THz TDS. In the gas-phase, flames, plumes, and explosive vapor are of greatest interest. Many explosives carry unique spectral fingerprints in the THz range, and have been detected in the gas-phase, one example being 2,4-dinitrotoluene (DNT) [107]. Also being studied are seemingly innocuous gas-phase bomb components such as that of a propane-air flame consisting of H_2_O, NH_3_, and CH [108]. The flame itself may be detected and imaged thanks to ultrafast pulses. Recognition of variations in the duration of time it takes the pulse to traverse the flame as a function of density of the heated air allow for such techniques [64].

THz TDS of water vapor is well established and has been useful in distinguishing subtle changes in the dielectric constant and thus water content of some samples [98,109]. Generally, however, the strong absorption of THz radiation in water vapor has proven to be an obstacle as the THz signal is significantly attenuated in ambient air. Historically, the solution to this drawback has been evacuating the system or purging it with nitrogen gas, limiting the applications of THz spectroscopy from a distance. Progress in ultrafast laser technology and processes have lent to the progress of this methodology with the development of filamentation methods [110,111].

Naturally, the application of THz TDS for gas sensing will continue to grow as data analysis algorithms improve. Yet this branch of THz spectroscopy has and will continue to owe much of its progress to the advancement of ultrafast lasers.

Though liquid-phase spectroscopy to date has been limited, the recent development of a detection scheme sensitive of pH-value shows promise for liquid spectroscopy as a growing application of THz TDS [112]. Like gases, information pertaining to different liquid species present in a mixture is often of great interest. Unique spectra of nitrobenzene in three different alkane solvents have been demonstrated [113]. Additionally, some work measuring the refractive index and absorption coefficient has further probed liquid dynamics [114].

**Figure 16.**
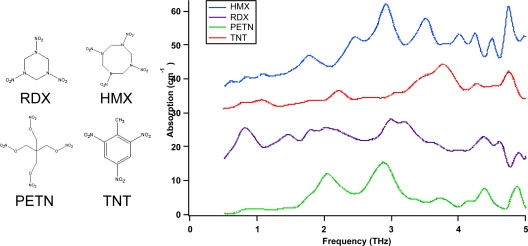
**[Left]** Chemical structure of the explosives RDX (1,3,5-trinitroperhydro-1,3, 5-triazine), HMX (1,3,5,7-tetranitroperhydro-1,3,5,7-tetrazocine), PETN (pentaerythritol tetranitrate), and TNT (2,4,6-trinitrotoluene). **[Right]** THz time-domain spectra of four explosives in polyethylene powder pellets. Data adapted from [85].

Solid phase THz spectroscopy has been used to investigate a wide variety of materials. As previously mentioned, explosives have received much attention due to unique spectral signatures (see Figure 16 below) differentiating many neat and plastic explosives from each other [84–92]. In many cases, the spectral features of the neat explosives in the THz region can be seen within the plastic explosive formulations, even when a combination of materials is present [115]. In addition to explosives, many benign materials simulating explosives in their visible characteristics have been studied for comparison. Sugars, e.g., lactose, sucrose, and marzipan, and drugs, e.g., co-codamol and aspirin, have been spectroscopically investigated to demonstrate the differences between the explosives and potential confusant materials [115]. For non-destructive imaging and evaluation, clothing materials and packing materials are also of interest [78,115,116]. Many clothing materials are primarily or at least partially transmissive to THz radiation. Many packing materials also have transmissive properties, including plastics and cardboard which would allow for non-destructive imaging of packages and containers. Although there are challenges and limitations for long stand-off applications using terahertz technology due to atmospheric attenuation and loss of signal due to reflective properties of the background substrate, there are plenty of opportunities for THz technology in the security arena. Teraview, Ltd. [117] has successfully demonstrated through-clothing detection of bulk explosives. Many security applications are short stand-off distance applications, where atmospheric attenuation does not pose significant difficulty.

Biomaterials and bio-systems have also been of significant interest for THz spectroscopy. Materials investigated include simple crystalline forms of amino acids, carbohydrates, and polypeptides [118] as well as short-chain polypeptides [119], and serine and cystine [120]. Much of the biological material work points to the extreme sensitivity to hydration of the structures as well as conformation.

## 5. Conclusions

We have presented three laser based stand-off sensing and spectroscopy techniques, which the introduction of ultrafast (femtosecond) laser technology has developed into new spectroscopy application not possible with traditional laser technology. One example is THz technology, a frequency range, which was, until a decade ago, almost inaccessible, requiring expensive sources and liquid Helium cooled bolometers. Now, portable systems using ultrafast fiber lasers for THz TDS are readily available. Similarly, frequency comb spectroscopy based on fs laser pulses might replace Fourier infrared spectroscopy and transform it into a standoff spectroscopic tool. CARS has overcome the low Raman cross-sections, allowing pollutants and explosives to be detected by their vibrational spectra, and with femtosecond laser technology, single-laser CARS can be as a viable standoff sensing tool.

The basic experimental technique for LIBS is quite simple and relates to established plasma emission spectroscopy techniques. Even so, the use of femtosecond lasers provides new avenues for developing this technique in remote sensing applications. In all its forms, LIBS inherently provides the ability to chemically analyze surfaces remotely, but femtosecond excitation can improve the quality of sampling by providing a more well-defined energy deposition process compared to nanosecond events. For bulk materials with complicated chemical compositions, this can simplify the relationship between the stoichiometry of the sample and that of the removed material. For detection of surface-borne species, femtosecond excitation increases the relative yield of surface material to that of the substrate leading to LIBS signals that can be more readily attributed to chemical species on the surface. Beyond these advantages, femtosecond excitation can produce LIBS signals more indicative of the sample since particular energy transfer mechanisms characteristic of the initial material state are primarily excited. Under nanosecond excitation, multiple energy transfer processes are acting and evolving simultaneously. Femtosecond irradiation is rapid compared to many energy exchange processes and the state of the system evolves without continually being altered by additional energy input. This provides interesting possibilities from a scientific point-of-view since it allows for a specificity of excitation that could yield LIBS signals more tightly correlated to particular chemical species. For example, tuning femtosecond pulse wavelength and shape might yield characteristic emission by the excited species. This approach has not been pursued but presents great opportunities for both experimental and fundamental theoretical studies. From a sensing perspective, femtosecond laser technology must improve considerably for application in the field. For the most part, current high energy, femtosecond systems suitable for performing LIBS under a variety of potential scenarios are laboratory-based instruments. An early report of femtosecond LIBS of a metal substrate at a range of 25 m was performed using a mobile laser system [121] however these types of systems require substantial support equipment. Fiber-based laser systems might provide the required pulse durations and portability for field application, however pulse energy and wavelength limitations currently preclude their use for effective LIBS sensing. However, given femtosecond laser technology evolution over the past two decades, suitable systems will certainly be available for future field use.

CARS spectroscopy has overcome the low Raman cross-sections, allowing pollutants and explosives to be detected by their vibrational spectra. Over the past decade, the emergence of femtosecond laser technology within this nonlinear spectroscopy has led to the development of single-laser CARS that extends the use of this technique to realistic detection applications that include standoff sensing of chemicals. Two technical developments that will have a clear impact on CARS applications in the future are the continued evolution of pulse shaping/coherent control and the inherent advantages of terawatt peak-power lasers for nonlinear spectroscopy. In regards to the former, the demonstrations of enhancing the molecular coupling between laser and materials via coherent pulse control have been quite encouraging. As this community continues to develop a deeper fundamental understanding of cause and effect between fs pulse profile and materials response, both theoretically and empirically, the development of applications such as stand-off detection and materials characterization will occur. In regards to benefits of high peak power fs lasers for this nonlinear technique, applications that can leverage the unique propagation characteristics of terawatt level sources, e.g., beam filamentation, can be expected to extend the distance over which pulse intensities can be maintained at levels that enable nonlinear interactions to occur.

Terahertz technology has greatly advanced due to the introduction and advancement of ultrashort femtosecond lasers. The ability to probe optical properties of materials, previously unavailable due to the lack of sources and detectors in this region of the spectrum, has generated much knowledge in the far-infrared region of the spectrum. THz technology is not without its own set of challenges, especially for stand-off applications. Recent advances in ultrafast laser technology, including fiber lasers, have provided commercial opportunities for portable time-domain spectroscopy and imaging systems, however, these systems still have the same limitations that many table-top systems have in low power emission from the THz source. Increasing the power output from THz sources as well as increasing the sensitivity of detection methods will greatly enhance the field as a whole. There are a multitude of research groups across the world actively working to solve these problems and advances in the technology are coming forward continually. One challenge that will limit the scope of the utility of terahertz technology is atmospheric attenuation of THz radiation. Although this limitation can be exploited in some applications, like medical imaging of skin cancer, it will overall limit the total distance the THz beam can travel for remote applications. THz generation in air can partially overcome this hurdle by generating and detecting the THz radiation at the target of interest, while propagating visible laser beams through the atmosphere, where attenuation is not a problem, however, this technique provides its own challenges with safety and control using high power laser beams that need precise control at large distances in order to appropriately exploit the technique. Terahertz technology cannot solve all spectroscopic and sensing problems, as no technology is the total solution to our sensing needs, however, it does provide the opportunity for unique spectroscopic investigations and sensing modalities that contribute to the overall problem space facing the science of sensing. Continued advancement of ultrafast pulsed laser technology will assist terahertz technology as a whole, providing more varied opportunities for the technology to flourish, especially as lasers become more compact and more powerful.

The introduction of ultrashort laser pulses into sensing and spectroscopy has not just provided simple growth for these technologies, but caused a paradigm shift and opened new parameter spaces for sensing and spectroscopy. And we can expect that the advances in ultrafast laser technology-smaller, higher power, shorter pulses, wider wavelength availability–will not only increase the capabilities of the technologies discussed, but generate new technologies using signatures for material identification presently only accessible in laboratories for future remote and standoff techniques.
